# Subdural Empyema in Pediatric Bacterial Meningitis: A Case Report

**DOI:** 10.7759/cureus.51401

**Published:** 2023-12-31

**Authors:** Hadeel Alosaimi, Khalid Aljohani, Thamer Alatawi, Ibrahim Alghabban, Faisal Alatawi, Abdulwahab Alduraibi, Danah Almithn, Ahmad Abdultawab

**Affiliations:** 1 General Practice, University of Tabuk, Tabuk, SAU; 2 General Practice, Qassim University, Qassim, SAU; 3 General Practice, King Faisal University, Al-Ahsa, SAU; 4 Emergency Medicine, Dallah Hospital, Riyadh, SAU

**Keywords:** leptomeningeal enhancement, subdural empyema, encephalitis, sinus disease, meningitis

## Abstract

Bacterial meningitis in pediatric populations presents a formidable challenge, necessitating careful evaluation and swift intervention. The clinical spectrum of pediatric bacterial meningitis requires a clear understanding, considering its diverse presentations, risk factors, and evolving diagnostic and therapeutic approaches. We present the case of an eight-year-old male who presented with an acute onset of fever, severe headache, and vomiting following an upper respiratory tract infection. A physical examination revealed meningeal irritation signs, altered consciousness, and focal seizures. Laboratory results showed elevated inflammatory markers, and cerebrospinal fluid analysis indicated abnormalities. Initial imaging displayed sinus involvement, but the patient's condition deteriorated. Subsequent magnetic resonance imaging revealed subdural empyema and meningoencephalitis. *Streptococcus pneumoniae* was identified as the causative agent. Subsequently, tailored antibiotic therapy and urgent neurosurgical interventions were initiated. The patient recovered with the resolution of neurological deficits. This case underscores the complexity of pediatric bacterial meningitis and its potential complications, emphasizing the relationship between upper respiratory tract infections, sinus involvement, and meningitis development. A multidisciplinary approach, combining targeted antimicrobial therapy with neurosurgical intervention, proved crucial for optimal management and favorable outcomes. This detailed case report highlights the importance of early diagnosis and comprehensive management in pediatric bacterial meningitis cases.

## Introduction

Bacterial meningitis in pediatric populations poses a complex and potentially life-threatening challenge, requiring careful clinical evaluation and prompt intervention. This inflammatory condition, affecting the protective membranes surrounding the brain and spinal cord, is frequently associated with various bacterial pathogens, among which *Streptococcus pneumoniae* stands out as a notable contributor [1]. The clinical presentation of pediatric bacterial meningitis can range from subtle symptoms to severe neurological compromise, necessitating a nuanced understanding of the disease's etiology, risk factors, and evolving diagnostic and therapeutic strategies. While advancements in vaccination have significantly reduced the incidence of certain bacterial strains causing meningitis, challenges persist in managing cases that present with complications such as subdural empyema and encephalitis [1,2]. This case report sheds light on a clinical scenario involving an eight-year-old male who presented with symptoms of bacterial meningitis subsequent to an upper respiratory tract infection. *Streptococcus pneumoniae*, a well-recognized pathogen in such cases, was identified as the causative agent, initiating a cascade of events that led to the development of subdural empyema and encephalitis. This report contributes to the existing medical literature by highlighting the complexity of pediatric bacterial meningitis, the potential implications of sinus involvement, and the pivotal role of advanced imaging techniques in guiding clinical decision-making for optimal patient outcomes.

## Case presentation

An eight-year-old male presented to the emergency department with an acute onset of high-grade fever, severe headache, and vomiting. The patient's initial physical examination revealed signs of meningeal irritation, including photophobia, neck stiffness, and positive Brudzinski and Kernig signs. Neurological examination revealed altered consciousness, with a Glasgow Coma Scale score of 11, signifying the severity of the patient's cognitive impairment.

Upon admission, the patient's vital signs showed a temperature of 38.9°C, a heart rate of 140 beats per minute, a respiratory rate of 26 breaths per minute, and blood pressure within the normal range for his age. Laboratory investigations revealed an elevated white blood cell count (15,000 cells/μL) and an elevated C-reactive protein level (18 mg/dL). The initial lumbar puncture demonstrated cerebrospinal fluid abnormalities, including an elevated white blood cell count (800 cells/μL, predominantly neutrophils), an elevated protein concentration (110 mg/dL), and a decreased glucose concentration (25 mg/dL). Empiric intravenous ceftriaxone and vancomycin were promptly initiated (Table 1).

**Table 1:** Initial hematological investigations.

Blood Investigations	Value	Normal Range	Indicator
White Blood Cell Count	15,000/μL	4,000 to 11,000/μL	High
Hemoglobin	13 g/dL	12 to 16 g/dL	Within Range
Platelet Count	200,000/μL	150,000 to 450,000/μL	Within Range
Blood Glucose	90 mg/dL	70 to 100 mg/dL	Within Range
Alanine Aminotransferase (ALT)	20 U/L	7 to 56 U/L	Within Range
Aspartate Aminotransferase (AST)	25 U/L	10 to 40 U/L	Within Range
Blood Urea Nitrogen (BUN)	15 mg/dL	7 to 20 mg/dL	Within Range
Creatinine	0.8 mg/dL	0.6 to 1.2 mg/dL	Within Range

The patient's medical history is notable for a recent upper respiratory tract infection, occurring one week before presentation, which serves as a pertinent precursor to the subsequent development of meningitis. Exploring the patient's medical history further revealed no significant predisposing factors, a familial history of immunodeficiency, or recent travel to regions with known infectious outbreaks.

**Figure 1:**
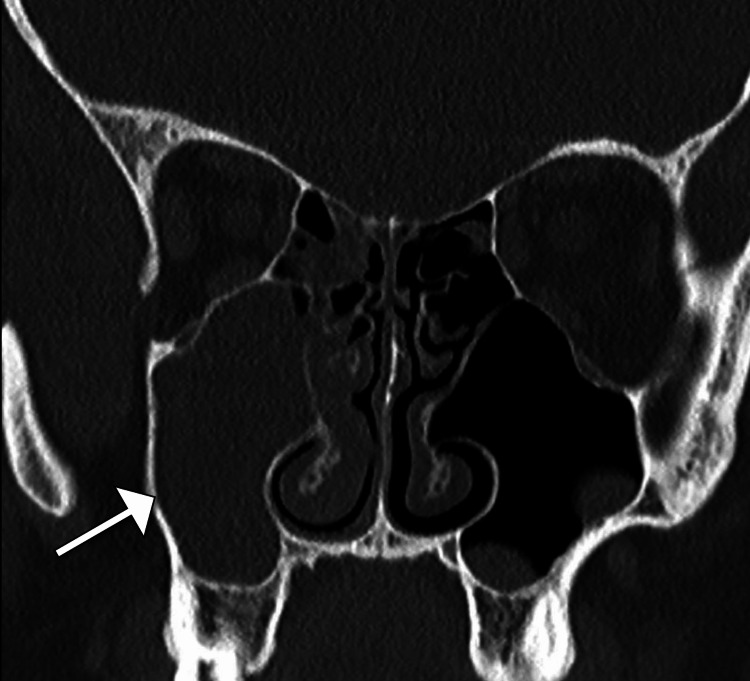
Coronal head CT image illustrating complete opacification of the right maxillary sinus (arrow), indicative of acute sinusitis. CT: computed tomography

**Figure 2:**
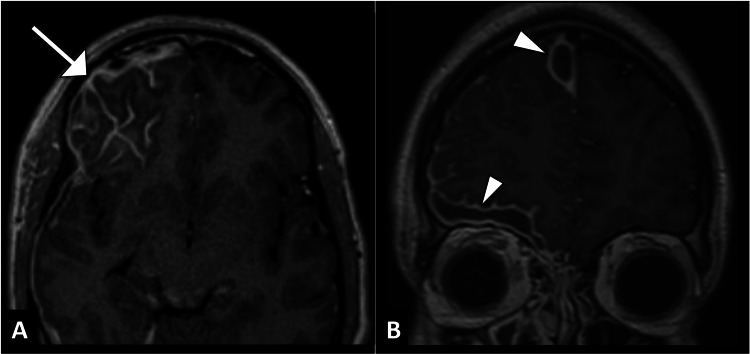
: Axial (A) and coronal (B) post-contrast brain MRI images highlight rim-enhancing lesions (arrowhead) and leptomeningeal enhancement (arrow), confirming the presence of subdural empyema in the context of meningoencephalitis. MRI: magnetic resonance imaging

An initial computed tomography scan of the head revealed mucosal thickening of the right maxillary and ethmoid sinuses, causing near-complete opacification, but no abnormalities in the brain parenchyma were noted (Figure 1). However, the patient's clinical condition continued to deteriorate over the next few days. Given the worsening symptoms, magnetic resonance imaging was performed, revealing multiple rim-enhancing subdural collections along the right parafalcine region and at the floor of the anterior cranial fossa, indicative of subdural empyema (Figure 2).

Initially, the causative organism was suspected to be *Streptococcus pneumoniae*. The patient's management was adjusted to include continued intravenous antibiotics, and a neurosurgical consultation was urgently sought. The decision was made to proceed with a burr hole drainage procedure to evacuate the subdural empyema. Intraoperatively, the purulent material was successfully drained. Confirmation of the causative organism was obtained through culture and sensitivity analysis of purulent material during the burr hole drainage procedure.

The hospital course was complicated by ongoing seizures, necessitating adjustments in anti-seizure medications. Following the burr hole drainage procedure and continued antibiotic therapy, the patient's laboratory investigations before discharge demonstrated significant improvement. Notably, the white blood cell count decreased to 8,000 cells/μL. Clinical examination at this stage revealed the resolution of meningeal signs and improved neurological status. Subsequent outpatient follow-up appointments revealed a complete recovery, with the resolution of neurological deficits.

## Discussion

Meningitis is a serious medical condition characterized by inflammation of the meninges, the protective membranes surrounding the brain and spinal cord. It can be caused by various infectious agents, including bacteria, viruses, fungi, and, rarely, parasites [1]. The multifaceted presentation of bacterial meningitis in pediatric patients, as illuminated by this case, underscores the intricate nature of *Streptococcus pneumoniae* infections and their potential for grave complications. The paramount significance of this case lies in its revelation of the nexus between upper respiratory tract infections, sinus involvement, and the subsequent development of meningitis and its complications.

The clinical presentation of meningitis is diverse and can include symptoms such as fever, headache, neck stiffness, photophobia, and altered mental status. The onset and progression of symptoms may vary depending on the causative agent, making an accurate and prompt diagnosis challenging [2,3].

Meningitis can lead to a spectrum of complications, both acute and long-term [1-3]. Acute complications include septic shock, cerebral edema, and hydrocephalus, while long-term complications encompass neurological deficits, hearing loss, and cognitive impairment. Meningitis-associated empyema, a purulent collection within the subdural or epidural space, emerges as a severe complication of meningitis, often complicating the clinical course and increasing the risk of morbidity and mortality [3,4].

Subdural empyema, as observed in this pediatric meningitis case, is considered a particularly severe complication due to the potential for rapid neurological deterioration and increased morbidity. In children, the confined space within the cranial vault can lead to elevated intracranial pressure, further emphasizing the critical nature of this complication [3,4]. Clinicians should maintain a high index of suspicion for this complication in cases of worsening neurological symptoms, persistent fever, or signs of meningeal irritation. Routine neuroimaging, including magnetic resonance imaging, can aid in early detection, allowing for timely intervention and potentially mitigating the progression to severe complications [3-5].

The management of meningitis-associated empyema requires a multidisciplinary approach. Antibiotic therapy targeting the causative pathogen is fundamental, and in some cases, surgical intervention may be necessary to evacuate purulent collections [2-4]. The successful outcome of this case underscores the importance of prompt and comprehensive management, combining targeted antimicrobial therapy with neurosurgical intervention for the evacuation of subdural empyema.

## Conclusions

In conclusion, this detailed case report illuminates the clinical trajectory of a pediatric patient with *Streptococcus pneumoniae* bacterial meningitis, complicated by subdural empyema and encephalitis. The observed association between upper respiratory tract infections, bacterial meningitis, and the ensuing intracranial complications underscores the need for a comprehensive evaluation of extracranial sources in pediatric meningitis cases. Our findings emphasize the critical role of advanced imaging, specifically magnetic resonance imaging, in guiding precise diagnosis and therapeutic interventions. The successful outcome achieved through tailored antimicrobial therapy and neurosurgical intervention highlights the importance of prompt diagnosis and management of these cases. The key takeaway from this case is the imperative for heightened vigilance among medical practitioners when confronted with pediatric patients presenting an acute febrile illness after an upper respiratory tract infection. The development of subdural empyema emphasizes the potential severity of meningitis complications. Incorporating routine neuroimaging early in the diagnostic process is essential for timely intervention and preventing further neurological deterioration. 

## References

[1] Agrawal S, Nadel S (2011). Acute bacterial meningitis in infants and children: epidemiology and management. Paediatr Drugs.

[2] Curtis S, Stobart K, Vandermeer B, Simel DL, Klassen T (2010). Clinical features suggestive of meningitis in children: a systematic review of prospective data. Pediatrics.

[3] Teixeira DC, Diniz LM, Guimarães NS, Moreira HM, Teixeira CC, Romanelli RM (2020). Risk factors associated with the outcomes of pediatric bacterial meningitis: a systematic review. J Pediatr (Rio J).

[4] Tacon CL, Flower O (2012). Diagnosis and management of bacterial meningitis in the paediatric population: a review. Emerg Med Int.

[5] Alamarat Z, Hasbun R (2020). Management of acute bacterial meningitis in children. Infect Drug Resist.

